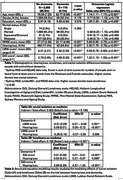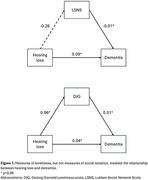# Loneliness partially mediates the relationship between hearing loss and all‐cause dementia: Preliminary findings from the COSMIC consortium

**DOI:** 10.1002/alz70858_106323

**Published:** 2025-12-24

**Authors:** Russell J Chander, Darren M. Lipnicki, Ashleigh S. Vella, Rory Chen, Anbupalam Thalamuthu, Nikolaos Scarmeas, Martin P.J. van Boxtel, Sebastian Köhler, Kay Deckers, Jacobijn Gussekloo, Henry Brodaty, Perminder S. Sachdev, COSMIC consortium

**Affiliations:** ^1^ Centre for Healthy Brain Ageing (CHeBA), University of New South Wales, UNSW Sydney, NSW, Australia; ^2^ Centre for Healthy Brain Ageing (CHeBA), UNSW Sydney, Sydney, NSW, Australia; ^3^ National and Kapodistrian University of Athens, Athens, Greece; ^4^ School for Mental Health and Neuroscience, Maastricht University, Maastricht, Netherlands; ^5^ Department of Psychiatry and Neuropsychology, Alzheimer Centrum Limburg, Mental Health and Neuroscience Research Institute (MHeNs), Maastricht University, Maastricht, Netherlands; ^6^ Department of Public Health and Primary Care, V0‐6. PO Box 9600, 2300 RC, Leiden, Netherlands; ^7^ Neuropsychiatric Institute, Prince of Wales Hospital, Randwick, NSW, Australia

## Abstract

**Background:**

Social isolation and hearing loss are both crucial modifiable risk factors of dementia. It is possible that hearing loss renders a risk of reduced social contact due to lifestyle modifications and accessibility issues, thereby inextricably linking these risk factors. Additionally, loneliness, as a subjective appraisal of one's social connectedness, may be the more important factor in the relationship between hearing loss and dementia. This highlights the need to continually address social isolation and/or loneliness in people with hearing impairments in preventing dementia. Possible mediation effects of social isolation and loneliness on the relationship between hearing loss and dementia warrant investigation.

**Methods:**

Data from four community‐based ageing cohorts (two from the Netherlands, one from Greece, one from Australia) from the Cohort Studies of Memory in an International Consortium (COSMIC) were harmonized. All‐cause dementia status was determined by clinician or consensus diagnosis. Hearing loss was classified via self‐reported difficulties or current use of hearing aids. Social isolation was indexed by the Lubben Social Network Scale (LSNS) via the sum score of the 'number of people interacted with monthly' questions in the Relatives and Friends subscales. Loneliness was indexed by the 11‐item DeJong Gierveld Loneliness Scale (DJG).

**Results:**

There were 2,775 individuals aged 65 to 98 years [mean age 76.33 (SD 7.35) years], of which 58.2% were female. 115 (4.1%) had dementia at time of assessment. Compared to people with no dementia, people with dementia were older, had higher proportion of hearing loss, better LSNS scores, and poorer DJG scores. While hearing loss and LSNS were both independently associated with dementia, structural equation modelling showed no mediation effects. DJG scores were independently associated with dementia and had a partial mediation effect on the relationship between hearing loss and dementia, with the indirect effect making up 19% of the total effect.

**Conclusion:**

Social isolation, loneliness, and hearing loss were independently associated with dementia. Loneliness partially mediated the relationship between hearing loss and dementia. Further analysis is underway with additional studies from COSMIC, where longitudinal analysis with incident dementia will be conducted and the interactions between social isolation and loneliness will also be studied.